# Surgery

**Published:** 1897-06

**Authors:** Charles A. Morton


### SURGERY.

In the treatment of **paralytic club-foot**, a new method has been introduced into surgical practice in the last two years. The tendon of a paralysed muscle has been sewn to a portion of an active muscle. Dr. Milliken of New York in 1895 recorded[4] what he regarded as the first operation of the kind. The tibialis anticus was paralysed and the foot became much everted in flexion, but this condition was remedied by joining the tibialis anticus tendon to that of the extensor proprius pollicis which was active. Passive movement was begun early after the junction. A later communication from Dr. Milliken[5] gives a record of fourteen operations performed upon nine patients. In one case two-thirds of the sartorius was transplanted into the sheath of the paralysed quadriceps extensor of the thigh. The first attempt

[4] *Med. Rec.*, 1895, xlviii. 581. [5] *Ibid.*, 1896, l. 771.

failed, owing to contraction of the hamstrings, causing too much tension on the junction. A second attempt gave a good result. The author recommends transplantation of the whole sartorius in the future. Grafting of the extensor pollicis to the tendon of the paralysed tibialis anticus was performed in five cases. All the cases have been followed, and Milliken considers it proved that the extensor proprius pollicis can carry on the work of the paralysed tibialis anticus. The gastrocnemius was attached to the paralysed peroneus longus and brevis in two cases. The patients were kept under observation for over twelve months, and the apparatus in each case was left off and the patient's walk greatly improved. In one case the extensor longus digitorum was attached to the paralysed tibialis anticus, with decided improvement in the position of the foot when examined three months after the operation. In another case the reverse was done—the paralysed extensor longus digitorum was attached to the active tibialis anticus. The result in this case is not given. In one case the extensor proprius pollicis was attached to the paralysed extensor longus digitorum. In this case also the result is not given. In these two cases the gastrocnemius was also attached to the paralysed peronei, as already described. In only one case was tendon-grafting done in the upper limb. In this case a graft was taken from the deltoid muscle and attached to the paralysed triceps, and it seemed to take on its action fairly well. The author considers that when a whole group of muscles is paralysed, a healthy muscle must be transplanted, and given the insertion of the paralysed group: and that when only some muscles in the group are involved tendon grafting must be performed. He uses kangaroo tendon for suturing the tendons and their sheaths, and lays stress on the need for careful suturing of the latter as well as of the tendons. He points out that the best results of such operative procedures can be obtained only in young subjects. Winkelmann has also performed tendon-grafting for paralytic club-foot.[1] He detached a portion of the gastrocnemius muscle and fixed to it the tendon of the paralysed peronei, with excellent result.

The treatment of intractable **congenital talipes equino-varus** has been much discussed during the last few years. In 1892 Mr. Walsham opened a discussion on this important subject at the Annual Meeting of the British Medical Association,[2] and more lately in his book[3] he has again considered the question. Mr. Tubby has devoted much space[4] to its elucidation, and several papers have appeared in periodical literature, to which reference will be made. A discussion on the subject at a meeting of the British Orthopædic Society in 1895 is also of interest.

[1] *Deformities*, by A. H. Tubby, 1896, p. 450. [2] *Brit. M. J.*, 1893, i. 339.
[3] *The Deformities of the Human Foot*, by W. J. Walsham and W. K. Hughes, 1895.
[4] *Op. cit.*

In the less severe forms of the deformity simple subcutaneous tenotomy, or tenotomy combined with division of the ligaments at the inner side of the foot (syndesmotomy or Parker's operation), will allow of rectification of the position of the foot. But in severe cases something more is needed, and the question which occupies so much attention at the present time is, What is the best treatment for these? When we have once performed an open division of the contracted tissues (Phelps's operation) in a severe case, we begin to wonder how mere division of the tibial tendons, and even of the calcaneo-scaphoid ligaments and the anterior fasciculus of the deltoid ligament, can ever allow proper rectification of the deformity. The more structures we cut the more contracted tissues we find beyond them. Muscles and flexor tendons have to be divided before the foot can be unfolded from its varus position. If we try to save the long flexor tendons after division of the abductor pollicis and the short flexor digitorum, we find the toes drawn down by them, as a bow is bent by its string, and they have to be divided also. But many surgeons now hold that all these parts can be stretched sufficiently to allow of restoration of the foot to a good position by either the hand or some form of instrumental wrench, under an anæsthetic, and they advocate this bloodless method in preference to a free open division of the tissues, or removal of some of the tarsal bones or a wedge-shaped portion of the tarsus.

Mr. Robert Jones of Liverpool, who has done a large amount of orthopædic work, strongly advocates[1] **wrenching the foot** into position, and has reported a number of what (from the pictures) appear to be very good results after it. He uses a wrench known as Thomas's. Mr. Jones says he has performed excision of the astragalus in six cases and wedge-shaped tarsectomy thirteen times, with fairly satisfactory results, but that he has discarded these methods for forcible wrenching. He felt certain that all cases of club-foot could be successfully treated by Thomas's wrench, and that the failures that were reported were due to the fact that surgeons often did not learn its method of application, which he describes in detail. This method of treating intractable talipes equino-varus is also highly recommended by Mr. Tubby, who says that he considers the time is not far distant when the severe varieties of the deformity will be submitted to forcible rectification in all cases. Some surgeons perform tenotomy of the tibial tendons, tendo Achillis, and plantar fascia before wrenching, and begin with manual manipulation, and only employ the wrench if not successful with the hand. Sometimes, as might be expected, the skin gives way on the inner side of the foot during the wrenching, and hence it must be performed with antiseptic precautions, as if we were about to do a cutting operation. Gangrene appears only to follow wrenching if the foot has been too tightly put up

[1] *Tr. Brit. Orthop. Soc.*, 1896, i. 20.

in plaster after, but sloughing has occurred from the pressure of the metal wrench.[1] When we remember the anatomy of talipes equino-varus—the enormous contraction of all the tissues on the inner two-thirds of the sole, and the marked changes in the bones, together with the fixation of the foot in its deformed position—it seems to me almost impossible that wrenching which falls short of almost tearing the foot open can overcome such extreme resistance. It seems to be generally admitted that wrenching requires a much longer time than tarsectomy or Phelps's operation for rectification of the foot, and has often to be repeated under an anæsthetic. As to the amount of pain after the wrenching, there seems to be some difference of opinion.[2] Mr. Walsham, who as surgeon in charge of the orthopædic department of St. Bartholomew's Hospital has had a large experience of club-foot, says[3] he has very seldom performed forcible rectification, and expresses the opinion that it is not only unnecessarily severe, but is lacking in the scientific principles which should guide us in our endeavours to cure the deformity. He states that a few cases have been reported in which it is said that after some years there has been no relapse.

**Phelps's operation** has now become well established. It has been warmly advocated by Mr. Edmund Owen in a paper read before the Royal Medical and Chirurgical Society in 1892[4] on the radical treatment of severe talipes equino-varus in children. Mr. Owen always first divides the tendo Achillis. As the structures divided in the operation are given so differently by surgical writers, I have referred to Phelps's original description of the operation published in America in 1881.[5] He says he carried the incision two-thirds across the sole, and divided the plantar fascia, the flexor brevis digitorum, the flexor longus digitorum, the flexor accessorius, the tibialis posticus, the flexor longus pollicis, flexor brevis pollicis, and the abductor pollicis. He states that he cut the artery and nerve in one case, but saved it in the other. This must refer to the internal plantar artery and nerve. All three patients were children. A very important paper by Dr. Phelps on his own operation has just been published.[6] He now frequently combines osteotomy of the neck of the astragalus, or even in some cases removal of a wedge-shaped piece of bone from the os calcis, with an open division of the soft parts on the inner aspect of the sole. In making this division of the soft parts he only makes the incision through the skin one quarter of the distance across the sole, starting from the inner malleolus, and through this incision divides the tibialis posticus, the abductor pollicis, the plantar fascia, the flexor brevis and longus digitorum, and the deltoid ligament. The tendo Achillis is divided subcutaneously. He

[1] Tubby, *op. cit.*, p. 418; and Walsham and Hughes, *op. cit.*, p. 175.
[2] *Tr. Brit. Orthop. Soc.*, 1896, i. 24, 28. [3] *Op. cit.*, p. 175.
[4] *Med.-Chir. Tr.*, 1893, lxxvi. 89. [5] *Med. Rec.*, 1881, xx. 337.
[6] *Post-Graduate*, 1897, xii. 195.

would only operate when the position of the foot cannot be corrected by manipulation with the hand or an instrumental wrench, and uses the wrench in conjunction with his open incision. He believes success lies in over-correcting the deformity in every case, and says if this is done relapse will probably not occur. He has not found a sensitive scar or flat-foot after his operations. Under one year of age he thinks tenotomy by the subcutaneous method will be sufficient; but he considers that if by manipulation the deformed position of the infant's foot cannot be rectified at the end of three months from birth, tenotomy should be performed. He agrees with most surgeons that treatment by manipulation and strapping should be commenced directly the child is born. He has now performed the operation which he originally described, or the modified form of it, in some cases combined with osteotomy, 538 times. He seems to have had no mortality. It is disappointing that all he can tell us with regard to the question of relapse is that out of 140 cases he was able to trace after a year from the time of the operation, only ten had relapsed, and that the three cases on which he first operated, which I have already referred to, had not relapsed after a period of ten years. He admits that equino-varus after any operation or mechanical treatment is quite likely to relapse, and supervision by the surgeon will be required for months or years. He says: "In my travels through Germany I made casts of feet which had relapsed after these osteotomies in the hands of some of the most eminent and distinguished German surgeons, and the same observations are to be made in every country."

There is no doubt that with Phelps's first operation, the foot can be brought into very good position. I have done the operation several times, and have been very pleased with the result; but in order to obtain a really satisfactory position of the foot without tension, I have been obliged to divide a large number of structures in the sole, and for this purpose have had to carry the incision two-thirds across the sole, as Phelps originally did. Whenever I have started with the intention of only dividing the structures on the inner aspect, I have had to go on and divide the long and short flexors of the toes; but have never divided the external plantar artery and nerve, though I have often found that the plantar fascia over them was one of the resisting structures requiring division. I believe a free opening up of the astragalo-scaphoid joint is essential to success. An objection to Phelps's operation is that so many important structures are divided in the sole, but they do not seem to be essential in walking,[1] and a thoroughly plantigrade foot which has not been shortened by tarsectomy is a condition with which we may perhaps be satisfied, even if all its movements are not

1 Phelps (*loc. cit.*, p. 210), says that the motion of the toes was preserved in nearly all his cases, and in the few with loss of flexion of the toes locomotion seemed as perfect.

perfect. Another objection to the operation is that the contraction of the scar will tend to reproduce the deformity. Of course the free open division of Phelps's operation should only be made when subcutaneous tenotomy, or syndesmotomy, fails, or would obviously fail.

A method of filling up the gap in the astragalo-scaphoid joint by means of a wedge of decalcified bone, wired in position, was introduced a few years ago by Dr. William Gardner of Melbourne.[1] This will only keep the bones apart while the decalcified bone is being replaced by fibrous tissue, and this fibrous tissue must eventually contract just as if it had been formed from ordinary granulations. Mr. Kellock[2] refers to the tendency to relapse which he has seen after this operation when instrumental support is withdrawn. He thinks contraction in the scar may be avoided by transplanting a flap of the skin on the outer side of the foot (which becomes superfluous there when the deformity is corrected by Phelps's operation) on to the gap formed by division of the contracted tissues on the inner side. He first dissects up a flap of the skin, and a few days later transplants it, as in Croft's method for treating contracted cicatrices after burns.

Mr. Arbuthnot Lane[3] considers that there are only two satisfactory methods of treatment for severe forms of equino-varus—Phelps's operation, or a new method which he has introduced. In Buchanan's operation all the structures on the inner side of the sole are divided subcutaneously; but Lane divides all the structures right across the sole—vessels, nerves, tendons, &c.—but also subcutaneously. What advantage this can be I must say I fail to understand, and as he admits—and as indeed must be evident to anyone who has several times performed Phelps's operation—the foot cannot at first be placed in good position by reason of the non-division of the contracted skin. In connection with this method of operating I will again refer to Mr. Edmund Owen's paper.[4] He says: "However vigorously the knife may have been used in the anatomical darkness of a subcutaneous operation, the complete effacement of the deformity is effectually hindered by the skin, which is not only thick and resisting, but is closely connected with the subjacent plantar fascia." Mr. Walsham is not at all favourable to Mr. Lane's method.[5] He has seen relapsed cases after it, but relapse will of course occur after any method if the parents neglect to carry out the surgeon's instructions. I cannot conceive that it is possible that either after Phelps's operation or Lane's modification of it, the proximal and distal ends of the tendons unite across the gap filled by granulations, or organising blood-clot, in such a way that the tendons are ever restored in exact continuity, as separate tendons.

Mr. Arbuthnot Lane, in performing the ordinary Phelps

[1] *Austral. M. J.*, 1893, xv. 435. [2] *Lancet*, 1895, i. 805.
[3] *Lancet*, 1893, ii. 432. [4] *Loc. cit.* [5] *Op. cit.*, p. 214.

operation, has hastened the healing by placing a large skin graft on the wound on the second day.[1]

As to the relative advantages of Phelps's operation and **tarsectomy**, or the desirability of performing tarsectomy at all in children, much difference of opinion exists. Mr. Walsham[2] admits that the foot is left in an imperfect condition, but becomes a useful plantigrade foot. He generally excises the astragalus, but he says this alone is often not enough, and a portion of the os calcis or other tarsal bones or the external malleolus has to be removed. Mr. Tubby, on the other hand, thinks that tarsectomy can very seldom be required, but then he is in favour of wrenching, and he contrasts the condition of the "elastic" foot after wrenching with the foot after tarsectomy, which he points out is often shortened and inelastic, with imperfect movement at the ankle and mid-tarsal joints, but he admits that the period of treatment is much shortened by tarsectomy. Relapse he has seen in an extreme degree years after tarsectomy, and when this occurs he considers nothing further can be done. This is, however, not always so. I have lately had a relapsed case of severe talipes equino-varus after tarsectomy, in which I completely restored the foot to the plantigrade position by removing another wedge of bone. The case at the time of the first operation was not under my care. When we do a wedge-shaped tarsectomy we feel that the contraction of the scar-tissue will not tend to reproduce the deformity, as it does in Phelps's operation; and when we consider the enormous change which exists in the shape of the bones in most severe long-standing cases, it seems very unlikely that anything short of an operation which deals with them will ultimately succeed. I have myself performed wedge-shaped tarsectomy or Phelps's operation a dozen times for severe equino-varus, and at present I do not feel certain which is the best for all cases; I think each case must be judged by its anatomical conditions. The risks of tarsectomy are considered in a paper by Wilson.[3] He has collected the records of 435 cases, and finds there have been in this number seven deaths, three from septicæmia, three from diarrhœa, and one from carbolic acid poisoning. In one case the leg had to be amputated for gangrene. Astragalectomy was found to be more frequently practised than cuneiform tarsectomy. The performance of some form of tarsectomy in the severer forms of congenital talipes equino-varus is advocated by Hartley of New York in a paper read before the New York Surgical Society,[4] and by other American surgeons at the same meeting.[5] Hopkins of Philadelphia describes[6] a method of correcting inveterate talipes varus by the artificial production of Pott's fracture deformity. He resects about half-an-inch of the lower end of the fibula, and then forcibly abducts the foot until the

[1] *Loc. cit.* [2] *Op. cit.*, and *Brit. M. J.*, 1893, i. 339. [3] Tubby, *op. cit.*, p. 446. [4] *Ann. Surg.*, 1894, xix. 257. [5] *Ibid.*, 367 [6] *Ibid.*, 1895, xxi. 461.

deformity disappears. He has operated on three cases, and one case figured in his paper indicates a good result. If the deformity consisted simply in adduction of the whole foot, however marked, it would be easy to understand how such an operation would remedy it; but when we remember the twisting inwards of the front part of the foot at the mid-tarsal joint, it seems unlikely that an operation which can only allow of abnormal abduction at the ankle can give a satisfactory result.

CHARLES A. MORTON.

